# A New Method for Syndrome Classification of Non-Small-Cell Lung Cancer Based on Data of Tongue and Pulse with Machine Learning

**DOI:** 10.1155/2021/1337558

**Published:** 2021-08-11

**Authors:** Yu-lin Shi, Jia-yi Liu, Xiao-juan Hu, Li-ping Tu, Ji Cui, Jun Li, Zi-juan Bi, Jia-cai Li, Ling Xu, Jia-tuo Xu

**Affiliations:** ^1^Basic Medical College, Shanghai University of Traditional Chinese Medicine, 1200 Cailun Road, Pudong, Shanghai, China; ^2^Shanghai Innovation Center of TCM Health Service, Shanghai University of Traditional Chinese Medicine, 1200 Cailun Road, Pudong, Shanghai, China; ^3^Shanghai University of Traditional Chinese Medicine Yueyang Hospital of Integrated Traditional Chinese Medicine and Western Medicine, 110 Ganhe Road, Hongkou, Shanghai, China

## Abstract

**Objective:**

To explore the data characteristics of tongue and pulse of non-small-cell lung cancer with Qi deficiency syndrome and Yin deficiency syndrome, establish syndrome classification model based on data of tongue and pulse by using machine learning methods, and evaluate the feasibility of syndrome classification based on data of tongue and pulse.

**Methods:**

We collected tongue and pulse of non-small-cell lung cancer patients with Qi deficiency syndrome (*n* = 163), patients with Yin deficiency syndrome (*n* = 174), and healthy controls (*n* = 185) using intelligent tongue diagnosis analysis instrument and pulse diagnosis analysis instrument, respectively. We described the characteristics and examined the correlation of data of tongue and pulse. Four machine learning methods, namely, random forest, logistic regression, support vector machine, and neural network, were used to establish the classification models based on symptom, tongue and pulse, and symptom and tongue and pulse, respectively.

**Results:**

Significant difference indices of tongue diagnosis between Qi deficiency syndrome and Yin deficiency syndrome were TB-a, TB-S, TB-Cr, TC-a, TC-S, TC-Cr, perAll, and the tongue coating texture indices including TC-CON, TC-ASM, TC-MEAN, and TC-ENT. Significant difference indices of pulse diagnosis were t_4_ and t_5_. The classification performance of each model based on different datasets was as follows: tongue and pulse < symptom < symptom and tongue and pulse. The neural network model had a better classification performance for symptom and tongue and pulse datasets, with an area under the ROC curves and accuracy rate which were 0.9401 and 0.8806.

**Conclusions:**

It was feasible to use tongue data and pulse data as one of the objective diagnostic basis in Qi deficiency syndrome and Yin deficiency syndrome of non-small-cell lung cancer.

## 1. Introduction

Lung cancer is a common malignant tumor of the lung and is a major cause of morbidity and mortality. It is estimated that the number of deaths from lung cancer accounts for about 24% of all cancer deaths in the United States [1, 2]. An organization report shows that lung cancer causes approximately 1.76 million deaths worldwide each year, accounting for 18.7% of all cancer deaths [3]. Non-small-cell lung cancer (NSCLC) is the most common histological type of lung cancer, accounting for more than 80% of primary lung cancers [4]. Sixty percent of NSCLC cases have metastasized at the time of diagnosis. The 5-year survival rate for advanced NSCLC is lower than 5%, and early diagnosis of lung cancer is an important opportunity to reduce mortality [5, 6]. The current treatment methods for NSCLC mainly include surgery, radiotherapy, chemotherapy, and targeted therapy [7, 8]. Chemotherapy is the most common treatment. However, patients with poor health often have a low tolerance to conventional treatment with a tendency of drug resistance [9]. Traditional Chinese medicine (TCM) has a long history and rich experience in the treatment of lung cancer, which is one of the main methods of comprehensive treatment of lung cancer in China. Systematic evaluation of TCM shows that TCM combined with radiotherapy and chemotherapy and targeted therapy had certain advantages in alleviating symptoms, stabilizing tumors, improving life quality, and prolonging survival period [10]. TCM has been proved to be an effective method for the treatment of advanced lung cancer. On the basis of accurate syndrome differentiation, TCM plays an active role in each stage of the occurrence and development of lung cancer [11, 12].

Syndrome differentiation and treatment is the basic principle of TCM to diagnose and deal with diseases. It is a process of comprehensive judgment on the four types of diagnostic information of patients based on the theory of TCM combined with the doctor's experience [13]. Accurate syndrome differentiation is able to provide a basis for the treatment of diseases and is the foundation of clinical efficacy. Traditional syndrome differentiation and treatment inevitably suffer from subjectivity and ambiguity, which actually hinders the development of TCM. Microsyndrome differentiation is a method of using modern advanced technology to go deep into the body's microcosmic level to understand and differentiate syndromes on the basis of macroscopic syndrome differentiation. Microsyndrome differentiation can be used to guide disease differentiation and syndrome differentiation, explore the cause and pathogenesis, and evaluate the efficacy and guide the prognosis of the disease [14]. Previous studies have verified that there is a close relationship between different syndromes and physical and chemical indices. A combination of microindex and macrosymptom can assist syndrome differentiation effectively.

With the rapid development of modern research on tongue and pulse diagnoses, a variety of tongue and pulse diagnoses instruments are widely used in clinical practice. This has generated a large number of objective data of tongue and pulse diagnoses, which are also microscopic indices in a sense. In recent years, studies based on data of tongue and pulse diagnoses have been increasing, with many researchers applying machine learning and data mining methods to the fields of image recognition, target detection, natural language processing, and others [15–18]. In addition, studies have demonstrated that accurate detection, identification, and multidimensional quantitative analysis based on tongue data and pulse data have been gradually applied to disease diagnosis. By constructing the diagnostic relationship between tongue and pulse and health status, it not only saves medical resources but also greatly improves diagnosis efficiency and treatment [19–22]. Qi deficiency syndrome and Yin deficiency syndrome are the two main common syndromes of NSCLC. When the symptoms are not obvious, the traditional symptom-based syndrome differentiation cannot be carried out. The modern study of tongue and pulse diagnoses research provides a good data basis for TCM syndrome differentiation.

Tongue data and pulse data are the most representative data of four diagnoses of TCM. The data collected and analyzed under the standardized condition has a high level of stability, which provides reliable objective data for intelligent syndrome differentiation. Among all kinds of syndromes, tongue and pulse are related to some extent, but the traditional syndrome differentiation cannot be clearly explained due to the lack of accurate data. With the development of diagnosis technology, the analysis and interpretation of the relationship between tongue and pulse can be realized more clearly. In this study, two common syndromes of NSCLC were selected to explore the differences of tongue data and pulse data and quantitatively analyze the data correlation of tongue and pulse, using machine learning methods to establish syndrome classification models based on macrosymptom, objective tongue and pulse, and macrosymptom and objective tongue and pulse, and evaluate the contribution rate of the objective data of tongue and pulse to syndrome differentiation.

## 2. Materials and Methods

### 2.1. Study Design and Subjects

**Figure 1:**
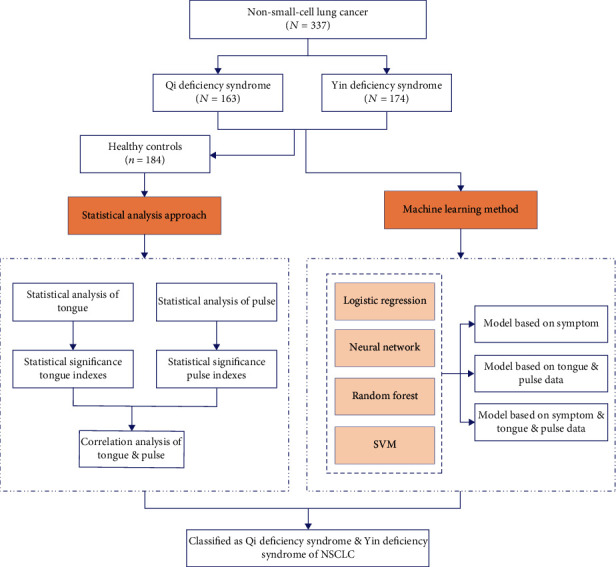
Flowchart.

We selected a total of 337 patients from the oncology department of Yueyang Hospital of Integrated Traditional Chinese and Western Medicine from January 2018 to October 2020, including 163 patients with Qi deficiency syndrome and 174 patients with Yin deficiency syndrome. All patients were pathologically or cytologically confirmed to be NSCLC. We additionally selected a total of 184 healthy people from Shuguang Hospital of Shanghai University of Traditional Chinese Medicine from January 2018 to October 2020 as the healthy controls. The flowchart is shown in Figure 1.

### 2.2. Diagnostic Criteria

Diagnostic criteria of Western medicine: according to the clinical practice guidelines for lung cancer screening issued by the National Comprehensive Cancer Network (NCCN) [23] and the fourth edition lung cancer histological classification standards of “Classification of Lung Tumors” [24, 25] issued by the World Health Organization.

TCM Syndrome Differentiation Standard: according to the “Technical Guidelines for Clinical Research of New Drugs of Syndromes” [26] and the Syndrome Part of TCM Clinical Diagnosis and Treatment Terms [27] and textbooks of Common Diseases and Symptoms in Internal Medicine of Traditional Chinese Medicine.

The main manifestations of Qi deficiency syndrome are cough, white or foamy phlegm, small amount of hemoptysis, chest tightness, shortness of breath, low fever, spontaneous sweating, lack of energy, pale complexion, poor appetite, loose stools, pale red tongue with tooth marks, thin white coating, and thin pulse. The main manifestations of Yin deficiency syndrome are cough without phlegm, or less but sticky phlegm, phlegm with blood, shortness of breath and dull chest pain, low fever, dry mouth, night sweat, upset and insomnia, red tongue, little or bare without tongue coating, and thin and rapid pulse. The syndrome was determined by at least three senior physicians to ensure the consistency and authenticity of syndrome differentiation.

### 2.3. Inclusion and Exclusion Criteria

The inclusion criteria are as follows: (1) meeting the above diagnostic criteria, (2) confirmed by pathology or cytology, (3) no serious liver or kidney damage, and (4) know and sign informed consent.

The exclusion criteria are as follows: (1) those who did not meet the inclusion criteria for NSCLC,(2) patients with Qi deficiency syndrome combined with Yin deficiency syndrome, (3) patients with severe primary diseases such as cardiovascular, cerebrovascular, liver, kidney, and blood system,(4) pregnant or lactating women, (5) psychopath, and (6) patients who were unable to cooperate with research work due to subjective and objective reasons and who had poor compliance.

### 2.4. Collecting Clinical Data of Tongue and Pulse

We used TFDA-1 digital tongue diagnosis instrument and PDA-1 digital pulse diagnosis instrument developed by the National Key Research and Development Program to collect tongue and pulse diagnostic data of patients, respectively. We used the Information Record Form of TCM Clinical Four Diagnostics (Copyright No.: 2016Z11L025702) developed by our research group to record the symptoms of patients [28]. All the work of tongue and pulse diagnoses collection and inquiry were completed by professional personnel of TCM or integrated TCM and western medicine who had received standardized training. Each patient was consulted by at least two professional researchers, and the syndromes of all patients were judged by three senior doctors to ensure the consistency and authenticity of data collection and interpretation and minimize deviation.

**Figure 2:**
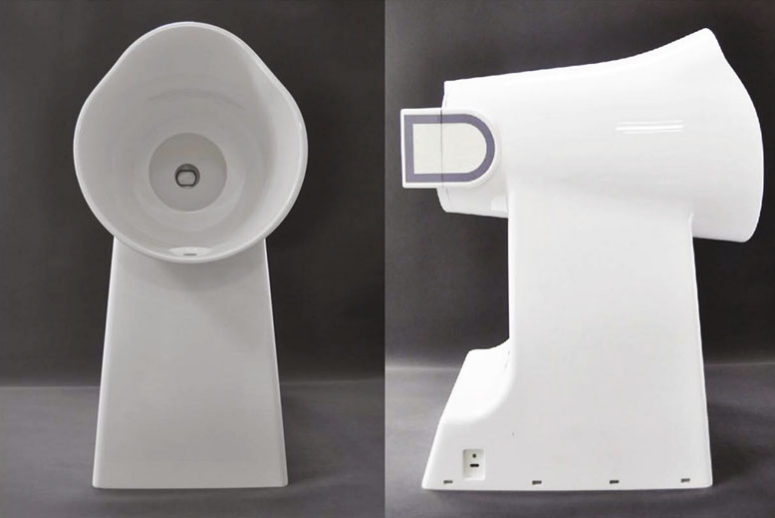
TFDA-1 digital tongue diagnosis instrument: (a) front view; (b) profile view.

**Figure 3:**
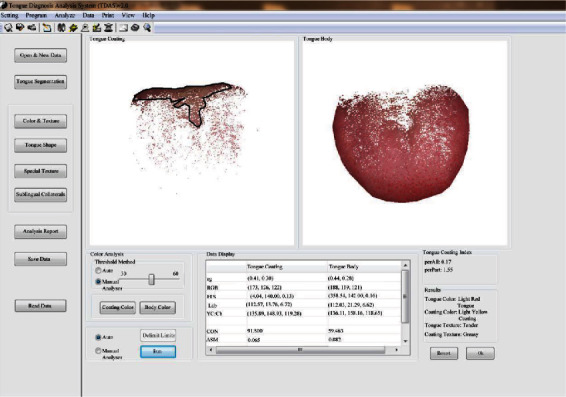
Tongue diagnosis analysis system (TDAS v2.0) of TFDA-1 digital tongue diagnosis instrument.

TFDA-1 digital tongue diagnosis instrument and tongue diagnosis analysis system (TDAS v2.0) are shown in Figures 2 and 3. The tongue was imaged by a video camera (Nikon 1 J5) with a fixed-focal lens which has 12 megapixels, and the picture resolution is 5568∗3712. TFDA-1 digital tongue diagnosis instrument uses LED light sources, and a curved reflector is set in front of the light sources to ensure the uniformity of illumination in all parts when the tongue image is collected. The color rendering index of light source is 96, and color temperature is around 5,000–6,500 K. Parameters of the TFDA-1 digital tongue diagnosis instrument are as follows: white balance, center-weighted metering, M mode, shutter speed of 1/125, aperture value of F6.3, and ISO sensitivity of 200.

**Figure 4:**
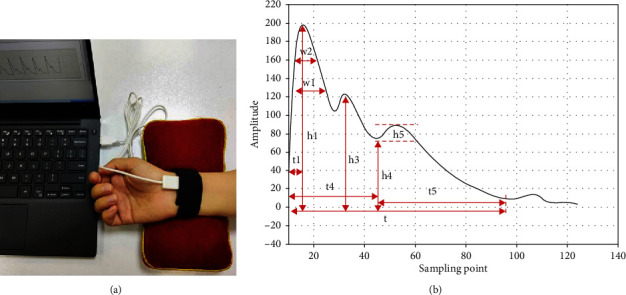
PDA-1 digital pulse diagnosis instrument and its corresponding sphygmogram: (a) PDA-1 digital pulse diagnosis instrument; (b) sphygmogram.

PDA-1 digital pulse diagnosis instrument and its corresponding sphygmogram are shown in Figure 4. The PDA-1 pulse diagnosis instrument uses a pressure sensor. Place the probe at the guan place of the patient's left hand, fix the strap, and adjust the tightness of the strap so that the sphygmogram reaches the best peak (the peak value of the main sphygmogram is 2 grids and above). Collect 30 s after the waveform is stable.

Tongue indices can be divided into two categories: tongue body (TB) index and tongue coating (TC) index which mainly come from the three color spaces of Lab, HIS, and YCrCb [29–32]. Each parameter of tongue diagnosis and pulse diagnosis has its corresponding medical significance [32–34]. In tongue indices, they are R (Red), G (Green), B (Blue), H (Hue), S(Saturation), I (Intensity) and L (Light), a (red-green axis), b (yellow-blue axis), Y (brightness), Cr (difference between red signal and brightness), Cb (difference between blue signal and brightness), texture indices include CON (Contrast), ASM (Angular Second Moment), ENT (Entropy), MEAN (Mean), and tongue coating indices include perAll and perPart. perAll represents the ratio of coated tongue area to total tongue area, and perPart represents the ratio of coated tongue area to noncoated tongue area. In pulse indices, *h*_1_-*h*_5_ mainly represent the amplitude height. *h*_1_ is the main wave amplitude, *h*_3_ is heavy wave front wave amplitude, *h*_3_/*h*_1_ is the ratio of heavy wave front wave amplitude to the amplitude of the main wave, *h*_4_ is the dicrotic notch amplitude, *h*_4_/*h*_1_ is the ratio of the dicrotic notch amplitude to the amplitude of the main wave, *h*_5_ is the gravity wave amplitude, and *h*_5_/*h*_1_ is the ratio of gravity wave amplitude to the amplitude to the amplitude of the main wave. *t* represents a complete pulse cycle, and *t*_1_ is the time value from the start point to the crest point of the main wave on the sphygmogram. *t*_4_ is the time value from the start point to the dicrotic notch on the sphygmogram, and *t*_5_ is the time value from the dicrotic notch to the end point on the sphygmogram. *w*_1_ is the width at 1/3 of the main wave, and *w*_2_ is the width at 1/5 of the main wave. All the tongue and pulse indices are extracted by special tongue analysis software (TDAS v2.0) and pulse analysis software (PulseCol).

### 2.5. Statistical Analysis

SPSS 26.0 was used for statistical analysis. Categorical variables were expressed as percentages (%). Continuous variables were expressed as mean ± standard deviation (SD) for those with normal distribution or median (interquartile range) for those with skewed distribution. Continuous variables were compared with analysis of variance (ANOVA) or rank-sum test (Kruskal-Wallis H test), and the correlation heat map was made by GraphPad Prism 8.0. A two-sided *P* value < 0.05 was considered statistically significant.

### 2.6. Classification by Machine Learning Approach

We used four machine learning methods, namely, neural network, random forest, support vector machine (SVM), and logistic regression to set the ratio of training set to test set at 8 : 2 using Orange (3.26.0) software. We used adjusted parameters of each model to establish classification and diagnosis models of Qi deficiency syndrome and Yin deficiency syndrome of NSCLC based on “symptom,” “tongue and pulse,” and “symptom and tongue and pulse”, respectively. We used accuracy, precision, F1-score (F1), sensitivity, specificity, and area under the curve (AUC) as evaluation indices to evaluate the predictive performance. AUC was the area under the ROC curve. The larger the value, the better the classification effect of the classifier. The calculation formula of each index was as follows:

(1) $$\begin{array}{c} \text{Accuracy}=\frac{\text{TP}+\text{TN}}{\text{TP}+\text{TN}+\text{FP}+\text{FN}}\times 100\%, \end{array}$$

(2) $$\begin{array}{c} \text{Precision}=\frac{\text{TP}}{\text{TP}+\text{FP}}\times 100\%, \end{array}$$

(3) $$\begin{array}{c} \text{Sensitivity}=\frac{\text{TP}}{\text{TP}+\text{FN}}\times 100\%, \end{array}$$

(4) $$\begin{array}{c} \text{Specificity}=\frac{\text{TN}}{\text{TN}+\text{FP}}\times 100\%, \end{array}$$

(5) $$\begin{array}{c} \text{F}1=\frac{2\times \text{Precision}\times \text{Sensitivity}}{\text{Precision}+\text{Sensitivity}}. \end{array}$$

In the above statements, True Positive (TP) was the positive sample predicted by the model as the positive category. True Negative (TN) was the negative sample predicted by the model as the negative category. False Positives (FP) was the negative sample predicted by the model as the positive category. False Negative (FN) was the positive sample predicted by the model as the negative category.

## 3. Results

### 3.1. Characteristics of Participants

The basic statistical analysis result of the three groups is shown in Table 1.

The result showed that people with Qi deficiency syndrome and Yin deficiency syndrome had a statistically significantly higher age than healthy controls. However, there was no difference in age between people with Qi deficiency syndrome and Yin deficiency syndrome.

### 3.2. Statistical Analysis of Tongue Data

Statistical analysis result of tongue diagnosis data in the three groups is shown in Table 2.

The result showed that (1) compared with Qi deficiency syndrome, there were more significant differences between Yin deficiency syndrome and the healthy controls. (2) In the significant difference indices between Yin deficiency syndrome and healthy controls, except for the texture index of tongue coating, the changes of tongue body index of Yin deficiency syndrome were more significant than that of tongue coating index. (3) Significant difference tongue indices between Qi deficiency syndrome and Yin deficiency syndrome were TB-a, TB-S, TB-Cr, TC-a, TC-S, TC-Cr, perAll, and TC-CON, TC-ASM, TC-MEAN, and TC-ENT; among them, TB-a, TB-Cr, TC-a, TC-S, TC-Cr, and TC-ASM of Yin deficiency syndrome were higher than those of Qi deficiency syndrome, while perAll, TC-CON, and TC-ENT of Yin deficiency syndrome were lower than those of Qi deficiency syndrome.

### 3.3. Statistical Analysis of Pulse Data

Statistical analysis result of pulse diagnosis data in the three groups is shown in Table 3.

The result showed that (1) the pulse parameters *t*_1_, *t*_4_, *t*_5_, *h*_1_, *h*_3_, *h*_4_, *h*_5_, *h*_1_/*t*_1_, *h*_4_/*h*_1_, *t*_4_/*t*_5_, *w*_1_/*t*, and *w*_2_/*t* of Qi deficiency syndrome and Yin deficiency syndrome had statistical significance compared with those of healthy controls. (2) Only two parameters, *t*_4_ and *t*_5_, showed statistically significant differences between Qi deficiency syndrome and Yin deficiency syndrome.

### 3.4. Correlation Analysis of Tongue Data and Pulse Data

**Figure 5:**
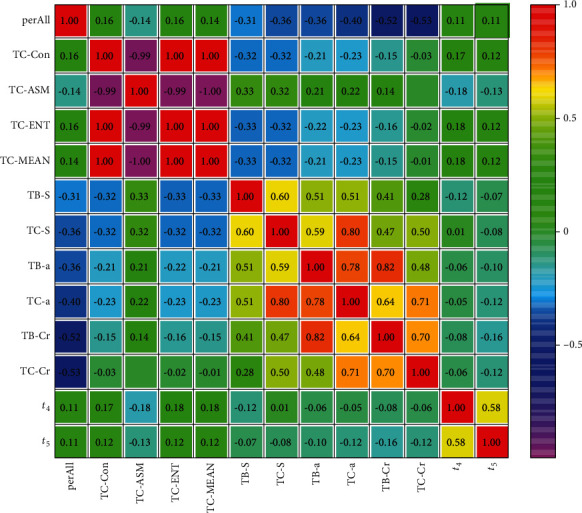
Heat map of tongue and pulse correlation analysis of Qi deficiency syndrome.

Tongue data and pulse data were statistically significantly correlated among people with Qi deficiency syndrome and Yin deficiency syndrome (Figure 5 and Table 4). Heat map result of Qi deficiency syndrome is shown in Figure 5.

Correlation analysis result of tongue data and pulse data between Qi deficiency syndrome is shown in Table 4.

The result showed that (1) there was a strong correlation between the tongue coating texture parameters, and the color space parameters of the tongue coating and the tongue body were also correlated. The correlation between the tongue coating texture parameters and the color space parameters was weaker than the correlation of the pulse parameters. (2) There was a definite correlation between pulse parameters *t*_4_ and tongue parameters TC-ASM, TC-ENT, and TC-MEAN, with a correlation coefficient of -0.18, 0.18, and 0.18, respectively. (3) There was a weak correlation between *t*_5_ and TB-Cr with a correlation coefficient of -0.16 (*P* < 0.05).

**Figure 6:**
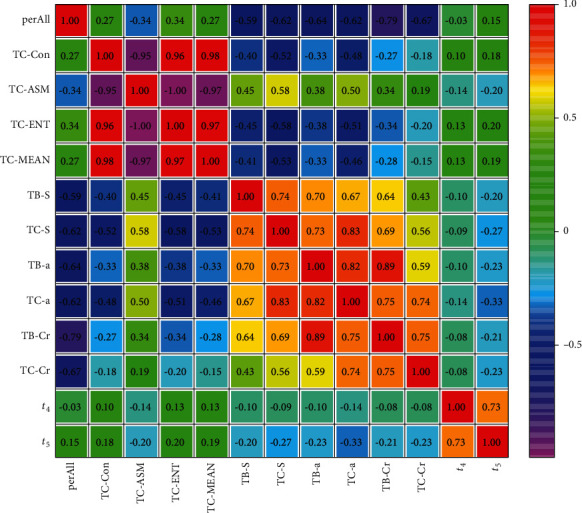
Heat map of tongue and pulse correlation analysis of Yin deficiency syndrome.

The heat map result of Yin deficiency syndrome is shown in Figure 6.

The correlation analysis result of tongue data and pulse data between Yin deficiency syndrome is shown in Table 5.

The result showed that (1) similar to Qi deficiency syndrome, the tongue coating texture parameters of Yin deficiency syndrome had a strong correlation, and the color space parameters of the tongue coating and tongue body were also strongly correlated. The correlation between tongue coating texture parameters and color space parameters was weaker than that of pulse parameters. (2) There was a certain correlation between pulse parameters *t*_4_ and tongue parameters TC-ASM and TC-a. Both of the correlation coefficients were -0.14, but the difference was not statistically significant (*P* > 0.05). (3) *t*_5_ was strongly correlated with TB-a, TC-S, TC-Cr, and TB-a, and the correlation coefficients were -0.33, -0.27, -0.23, and -0.23, respectively (*P* < 0.01). The correlation coefficients of *t*_5_ with TB-Cr, TB-S, and TC-ASM were -0.21, -0.20, and -0.20, respectively (*P* < 0.01).

The correlation analysis result showed that correlation intensity of tongue and pulse in Yin deficiency syndrome was significantly stronger than that in Qi deficiency syndrome, and compared with Qi deficiency syndrome, the correlation between *t*_4_ and tongue indices in Yin deficiency syndrome was significantly reduced, while the correlation between *t*_5_ and tongue indices was significantly increased.

### 3.5. Machine Learning Results

Based on neural network, random forest, SVM, and logistic regression four machine learning methods, the modeling result of Qi deficiency syndrome and Yin deficiency syndrome based on symptom, tongue and pulse, and symptom and tongue and pulse is shown in Table 6.

**Figure 7:**
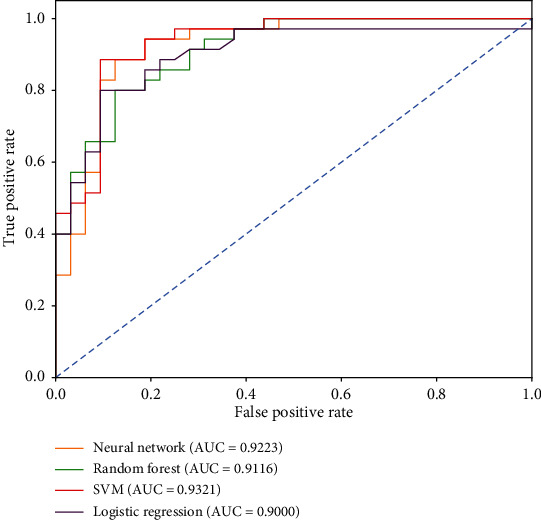
ROC curves of Qi deficiency syndrome model based on symptom.

**Figure 8:**
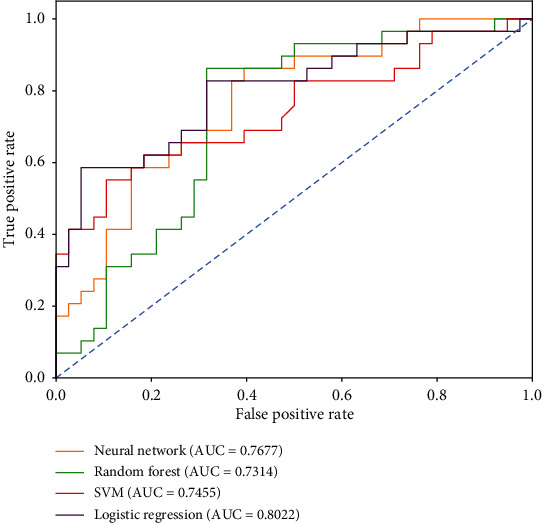
ROC curves of Qi deficiency syndrome model based on tongue and pulse.

**Figure 9:**
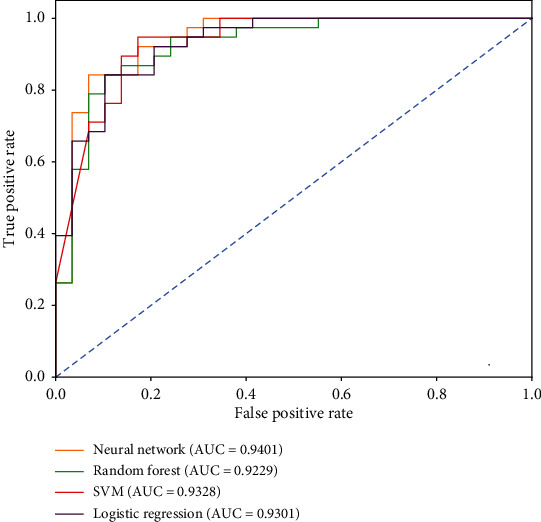
ROC curves of Qi deficiency syndrome model based on syndrome and tongue and pulse.

The ROC curves of the models based on symptom, tongue and pulse, and symptom and tongue and pulse are shown in Figures 7–9, respectively.

According to the above modeling results, the classification efficiency of each model based on different datasets had the following order: tongue and pulse < symptom < symptom and tongue and pulse. Among them, the SVM model had a better classification performance for symptom datasets, and the area under the ROC curve was 0.9321. The logistic regression model had a better classification performance for tongue and pulse datasets, with an area under the ROC curve of 0.9401. The neural network model had a better classification performance for the symptom and tongue and pulse datasets, with an area under the ROC curve of 0.9401.

## 4. Discussion

Treatment based on syndrome differentiation is the basic principle of TCM to recognize and treat diseases. It runs through the whole process of prevention and rehabilitation of medical care practices. Syndrome differentiation is used to recognize the disease and determine the syndrome, and treatment is to establish treatment methods and prescription drugs based on the results of syndrome differentiation. Syndrome differentiation is the prerequisite and basis for treatment. Accurate syndrome differentiation results in a good therapeutic effect. Qi deficiency syndrome and Yin deficiency syndrome are two common syndromes in TCM. According to the basic theory of TCM syndrome differentiation, Qi deficiency syndrome refers to the lack of vitality of the body and the decreased function of visceral organs. The main manifestations are fatigue, lack of energy, lazy speech, and weak pulse. Yin deficiency syndrome refers to the lack of Yin fluid in human body, its nourishing and nourishing functions are reduced, or Yin does not control Yang, and Yang is too hyperactive. The main manifestations are dry mouth and pharynx, dysphoria in chestpalms-soles, tidal fever, and night sweating. According to the principle of TCM syndrome differentiation and treatment, the principle and treatment method of Qi deficiency syndrome are to invigorate the spleen and replenish Qi, and the corresponding prescription is Sijunzi decoction. The principle and treatment method of Yin deficiency syndrome are to nourish Yin and clear lung, and the corresponding prescription is Shashen Maidong decoction.

### 4.1. Statistical Analysis of Tongue Data and Pulse Data of Qi Deficiency Syndrome and Yin Deficiency Syndrome

TCM is a promising and effective adjuvant therapy in the treatment of lung cancer. Compared with chemotherapy and radiotherapy, it has the advantages of availability, effectiveness, and low toxicity [35], although its various mechanisms deserve further study [36, 37]. In this study, the tongue parameters, including TB-a, TC-a, TB-Cr, and TC-Cr of Qi deficiency syndrome and Yin deficiency syndrome, represent the red value of tongue body and tongue coating. Larger values of tongue parameters reflect the redder or magenta tongue. In Yin deficiency syndrome, TB-a, TC-a, TB-Cr, and TC-Cr were all higher than those in Qi deficiency syndrome, indicating that the tongue of Yin deficiency syndrome was redder or magenta. S stands for saturation, and the higher the value of S, the brighter the tongue color will be. TC-S in Yin deficiency syndrome was higher than that in Qi deficiency syndrome, indicating that the tongue color of Yin deficiency syndrome was brighter. perAll is the ratio of tongue coating area to total tongue area. perAll has a higher diagnostic value for thick coating, and the higher the value, the thicker the tongue coating. perAll in Yin deficiency syndrome was lower than that in Qi deficiency syndrome, indicating that the tongue coating was thinner in Yin deficiency syndrome. Among the four parameters of texture parameters CON, ASM, ENT, and MEAN, the smaller the value of CON, ENT, and MEAN, the larger the ASM, reflecting that the more delicate the tongue texture or the more greasy the tongue coating. In this study, TC-CON and TC-ENT of Yin deficiency syndrome were significantly lower than those of Qi deficiency syndrome, while TC-ASM was higher than that of Qi deficiency syndrome, indicating that the tongue coating of Yin deficiency syndrome was greasier.

In the pulse parameters, *t*_4_ is the time value from the starting point to the descending isthmus of the sphygmogram, corresponding to the systolic period of left ventricle, and *t*_5_ is the time value from the dicrotic notch to the end point of the sphygmogram, corresponding to the diastolic period of left ventricle. *t*_4_ and *t*_5_ of Yin deficiency syndrome were smaller than those of Qi deficiency syndrome, indicating that the time of systole and diastole of Yin deficiency syndrome was shorter than those of Qi deficiency syndrome, and the pulsation cycle *t* of Yin deficiency syndrome also showed a decreasing trend, indicating that the pulse wave velocity of Yin deficiency syndrome was slightly higher. In addition, there was a phenomenon of elevation of Yin deficiency syndrome in dicrotic notch *h*_4_. Furthermore, indicrotic notch *h*_4_ in Yin deficiency syndrome was elevated. In the Qi deficiency syndrome, *h*_3_/*h*_1_, *h*_1_/*t*_1_, and *t*_1_ were prolonged, reflecting that the pulse force of the Qi deficiency syndrome was soft and weak, the amplitude of the main wave *h*_1_ was reduced, and the area under the sphygmogram was smaller, indicating that the pulse shape was thin and small. All in all, the tongue of Qi deficiency syndrome was pale and the pulse was weak, while the tongue body of Yin deficiency syndrome was more red or crimson, more brighter in tongue color, thinner and greasy in tongue coating, and more fine in pulse.

### 4.2. Modeling Analysis of Qi Deficiency Syndrome and Yin Deficiency Syndrome Based on Data of Tongue and Pulse

In recent years, with the rapid development of computer technology, different recognition algorithms and machine learning methods, such as logical regression [38], SVM [22, 39], random forest [40], and neural network [15, 41], and other data mining technologies have been widely used in medical research. The quantitative diagnosis of diagnostic information through various mathematical models has promoted the development of TCM informatization. In this study, symptom and tongue and pulse data were used to classify syndromes. The results showed that the classification efficiency of models based on different datasets was as follows: tongue and pulse < symptom < symptom and tongue and pulse, indicating that tongue and pulse data contributed to the classification of syndrome to some extent. Therefore, when faced with a complicated quantitative and qualitative, subjective and objective, determine and fuzzy, and massive TCM data combining linear and nonlinear, TCM syndrome associated with complex multidimensional characteristics and associated with multiple microindex, especially when symptoms were not evident, to explore the relationship between different syndromes and physical and chemical indices can effectively assist in syndrome differentiation. Research also showed that it was very reasonable to combine microindex with macrosymptoms. Using machine learning or data mining methods to build TCM syndrome or disease diagnosis model can make the process of syndrome differentiation and treatment more objective, standardized, and intelligent [42–44].

### 4.3. Limitations and Future Work

This research is based on the real-world investigation, and the results basically conform to the syndrome distribution feature of NSCLC in the clinic. However, there are also some limitations in the study. First of all, due to the limitation of time and place, the sample size of this study is not large enough. Secondly, the basic data statistics of subjects are not comprehensive enough, and there is a lack of statistics on height, weight, body mass index (BMI), history of present illness, past medical history, etc., which may affect the data results. Last but not the least, this study mainly focused on the common NSCLC syndrome of Qi deficiency and Yin deficiency, lacking more syndromes to explore. In the future, a large-scale and multicenter epidemiological investigation should be combined, the collection of four diagnostic information and basic characteristics needs to be more standardized and complete, and further researches based on more comprehensive syndrome differentiation results need to be carried out.

## 5. Conclusions

In conclusion, objective tongue and pulse data of NSCLC are useful for the classification of TCM syndrome, which can improve the accuracy of TCM syndrome classification to a certain extent. Tongue and pulse diagnosis parameters can provide new ideas and methods for TCM syndrome differentiation of Qi deficiency syndrome and Yin deficiency syndrome of NSCLC.

## Figures and Tables

**Table 1:** Basic statistical analysis.

Characteristic		Healthy controls (*n* = 184)	Qi deficiency syndrome (*n* = 163)	Yin deficiency syndrome (*n* = 174)
Sex, *n* (%)	Male	96 (52.17)	72 (44.17)	89 (51.15)
	Female	88 (47.83)	91 (55.83)	85 (48.85)
Age, years		27.00 (29.00-24.25)	67.00 (59.00-71.00)^∗∗^	67.00 (60.00-72.00)^∗∗^

vs. healthy controls, ^∗∗^*P* < 0.01.

**Table 2:** Statistical analysis of tongue diagnosis data (mean (SD), median (P_25_, P_75_)).

Domain	Color space	Index	Healthy controls (*n* = 184)	Qi deficiency syndrome (*n* = 163)	Yin deficiency syndrome (*n* = 174)
TB	Lab	TB-L	103.99 (100.81-108.79)	96.31 (75.15-102.89)^∗∗^	99.83 (80.51-103.24)^∗∗^
		TB-a	19.98 ± 2.82	19.31 ± 3.81	21.06 ± 4.23^∗^^##^
		TB-b	4.76 (0.82-7.00)	7.04 (5.47-8.28)^∗∗^	7.04 (5.47-8.28)^∗∗^
	HIS	TB-H	176.22 (168.50-180.95)	180.00 (177.98-182.83)^∗∗^	180.00 (177.98-182.83)^∗∗^
		TB-S	0.17 (0.16-0.20)	0.17 (0.15-0.19)	0.17 (0.15-0.19)^∗^^##^
		TB-I	117.00 (108.00-132.00)	116.00 (109.00-126.00)^∗∗^	116.00 (109.00-126.00)
	YCrCb	TB-Y	114.98 (107.03-126.56)	114.35 (106.900-123.72)	114.35 (106.900-123.72)^∗^
		TB-Cr	151.41 ± 3.05	152.29 ± 3.89	154.15 ± 4.44^∗∗^^##^
		TB-Cb	121.61 (119.75-124.82)	119.84 (118.53-120.99)^∗∗^	119.27 (118.09-120.57)^∗∗^
	Texture index	TB-CON	71.47 (46.96-99.54)	74.56 (48.28-94.64)	60.96 (45.32-86.08)
		TB-ASM	0.08 (0.07-0.10)	0.07 (0.07-0.09)	0.09 (0.07-0.10)
		TB-MEAN	0.03 (0.02-0.03)	0.03 (0.02-0.03)	0.02 (0.02-0.03)
		TB-ENT	1.21 (1.11-1.28)	1.22 (1.12-1.28)	1.17 (1.10-1.25)
TC	Lab	TC-L	109.24 (104.97-113.54)	89.38 (76.22-104.87)^∗∗^	95.35 (82.53-105.08)^∗∗^
		TC-a	12.31 ± 2.69	12.75 ± 3.21	14.25 ± 3.78^∗∗^^##^
		TC-b	2.71(-1.16-5.32)	5.59 (4.24-6.62)^∗∗^	5.86 (4.35-7.26)^∗∗^
	HIS	TC-H	176.70 (162.43-183.25)	183.00 (180.00-186.35)^∗∗^	182.58 (178.64-185.72)^∗∗^
		TC-S	0.11 (0.09-0.13)	0.12 (0.10-0.14)^∗^	0.13 (0.11-0.17)^∗∗^^##^
		TC-I	130.00 (117.00-142.75)	119.00 (99.00-135.00)^∗∗^	115.00 (92.75-133.00)^∗∗^
	YCrCb	TC-Y	126.78 (115.63-137.70)	118.65 (99.72-132.07)^∗∗^	114.02 (95.19-129.53)^∗∗^
		TC-Cr	142.89 (140.89-145.181)	143.97 (142.27-146.51)^∗∗^	145.49 (143.00-148.79)^∗∗^^##^
		TC-Cb	123.90 (121.54-127.61)	121.36 (120.34-122.81)^∗∗^	121.35 (120.01-122.67)^∗∗^
	Area index	perAll	0.54 (0.43-0.69)	0.44 (0.34-0.50)^∗∗^	0.38 (0.21-0.50)^∗∗^^#^
		perPart	1.09 (1.02-1.22)	1.24 (1.11-1.42)^∗∗^	1.28 (1.11-1.57)^∗∗^
	Texture index	TC-CON	89.27 (62.31-124.17)	83.13 (63.82-123.30)	71.53 (44.56-115.98)^∗∗^^##^
		TC-ASM	0.07 (0.06-0.08)	0.07 (0.06-0.08)	0.08 (0.06-0.10)^∗∗^^##^
		TC-MEAN	0.03 (0.02-0.03)	0.03 (0.02-0.03)	0.03 (0.02-0.03)^∗∗^^##^
		TC-ENT	1.26 (1.18-1.34)	1.25 (1.18-1.34)	1.21 (1.09-1.31)^∗∗^^##^

vs. healthy controls, ^∗^*P* < 0.05, vs. healthy controls, ^∗∗^*P* < 0.01. vs. Qi deficiency syndrome, ^#^*P* < 0.05, vs. Qi deficiency syndrome, ^##^*P* < 0.01.

**Table 3:** Statistical analysis of pulse diagnosis data (mean (SD), median (P_25_, P_75_)).

Index	Healthy controls (*n* = 184)	Qi deficiency syndrome (*n* = 163)	Yin deficiency syndrome (*n* = 174)
*t*_1_ (s)	0.13 (0.12-0.14)	0.14 (0.13-0.15)^∗∗^	0.14 (0.13-0.14)^∗∗^
*t*_4_ (s)	0.34 (0.32-0.36)	0.37 (0.35-0.39)^∗∗^	0.37 (0.34-0.39)^∗∗^^#^
*t*_5_ (s)	0.41 (0.39-0.42)	0.43 (0.41-0.46)^∗∗^	0.42 (0.40-0.44)^∗∗^^##^
*t* (s)	0.80 (0.75-0.88)	0.86 (0.76-0.97)^∗∗^	0.84 (0.72-0.94)
*h*_1_ (mv)	13.89 (11.53-16.41)	10.99 (7.62-15.42)^∗∗^	11.56 (8.86-16.51)^∗∗^
*h*_3_ (mv)	8.48 (6.56-10.59)	6.64 (4.38-10.07)^∗∗^	7.18 (4.85-10.12)^∗∗^
*h*_4_ (mv)	5.21 (4.18-6.32)	2.18 (1.37-3.24)^∗∗^	2.53 (1.44-3.50)^∗∗^
*h*_5_ (mv)	0.50 (0.15-0.95)	0.23 (0.05-0.69)^∗∗^	0.21 (0.05-0.60)^∗∗^
*h*_3_/*h*_1_	0.62 (0.52-0.70)	0.61 (0.53-0.71)	0.60 (0.49-0.73)
*h*_1_/*t*_1_	4.43 (3.49-5.35)	3.22 (2.26-4.57)^∗∗^	3.45 (2.68-4.82)^∗∗^
*h*_4_/*h*_1_	0.38 (0.32-0.43)	0.21 (0.12-0.31)^∗∗^	0.21 (0.14-0.28)^∗∗^
*t*_1_/*t*	0.16 (0.14-0.17)	0.16 (0.14-0.19)	0.17 (0.14-0.19)
*t*_4_/*t*_5_	0.83 (0.80-0.88)	0.86 (0.82-0.91)^∗∗^	0.87 (0.82-0.91)^∗∗^
*w*_1_/*t*	0.20 (0.15-0.23)	0.21 (0.19-0.23)^∗∗^	0.21 (0.19-0.23)^∗∗^
*w*_2_/*t*	0.12 (0.10-0.16)	0.15 (0.13-0.18)^∗∗^	0.15 (0.13-0.18)^∗∗^

vs. healthy controls, ^∗^*P* < 0.05, vs. healthy controls, ^∗∗^*P* < 0.01. vs. Qi deficiency syndrome, ^#^*P* < 0.05, vs. Qi deficiency syndrome, ^##^*P* < 0.01.

**Table 4:** Correlation analysis of tongue data and pulse data of Qi deficiency syndrome.

Index	perAll	TC-CON	TC-ASM	TC-ENT	TC-MEAN	TB-S	TC-S	TB-a	TC-a	TB-Cr	TC-Cr	*t*_4_	*t*_5_
perAll	1.00												
TC-CON	0.16^∗^	1.00											
TC-ASM	-0.14	-0.99^∗∗^	1.00										
TC-ENT	0.16^∗^	1.00^∗∗^	-0.99^∗∗^	1.00									
TC-MEAN	0.14	1.00^∗∗^	-1.00^∗∗^	1.00^∗∗^	1.00								
TB-S	-0.31^∗∗^	-0.32^∗∗^	0.33^∗∗^	-0.33^∗∗^	-0.33^∗∗^	1.00							
TC-S	-0.36^∗∗^	-0.32^∗∗^	0.32^∗∗^	-0.32^∗∗^	-0.32^∗∗^	0.60^∗∗^	1.00						
TB-a	-0.36^∗∗^	-0.21^∗∗^	0.21^∗∗^	-0.22^∗∗^	-0.21^∗∗^	0.51^∗∗^	0.59^∗∗^	1.00					
TC-a	-0.40^∗∗^	-0.23^∗∗^	0.22^∗∗^	-0.23^∗∗^	-0.23^∗∗^	0.51^∗∗^	0.80^∗∗^	0.78^∗∗^	1.00				
TB-Cr	-0.52^∗∗^	-0.15	0.14	-0.16^∗^	-0.15	0.41^∗∗^	0.47^∗∗^	0.82^∗∗^	0.64^∗∗^	1.00			
TC-Cr	-0.53^∗∗^	-0.03	-0.00	-0.02	-0.01	0.28^∗∗^	0.50^∗∗^	0.48^∗∗^	0.71^∗∗^	0.70^∗∗^	1.00		
*t*_4_	0.11	0.17^∗^	-0.18^∗^	0.18^∗^	0.18^∗^	-0.12	0.01	-0.06	-0.05	-0.08	-0.06	1.00	
*t*_5_	0.11	0.12	-0.13	0.12	0.12	-0.07	-0.08	-0.10	-0.13	-0.16^∗^	-0.12	0.58^∗∗^	1.00

^∗^*P* < 0.05, ^∗∗^*P* < 0.01.

**Table 5:** Correlation analysis of tongue data and pulse data of Yin deficiency syndrome.

Index	perAll	TC-CON	TC-ASM	TC-ENT	TC-MEAN	TB-S	TC-S	TB-a	TC-a	TB-Cr	TC-Cr	*t*_4_	*t*_5_
perAll	1.00												
TC-CON	0.27^∗∗^	1.00											
TC-ASM	-0.34^∗∗^	-0.95^∗∗^	1.00										
TC-ENT	0.34^∗∗^	0.96^∗∗^	-1.00^∗∗^	1.00									
TC-MEAN	0.27^∗∗^	0.98^∗∗^	-0.97^∗∗^	0.97^∗∗^	1.00								
TB-S	-0.59^∗∗^	-0.40^∗∗^	0.45^∗∗^	-0.45^∗∗^	-0.41^∗∗^	1.00							
TC-S	-0.62^∗∗^	-0.52^∗∗^	0.57^∗∗^	-0.58^∗∗^	-0.53^∗∗^	0.75^∗∗^	1.00						
TB-a	-0.64^∗∗^	-0.33^∗∗^	0.38^∗∗^	-0.39^∗∗^	-0.33^∗∗^	0.70^∗∗^	0.73^∗∗^	1.00					
TC-a	-0.62^∗∗^	-0.48^∗∗^	0.50^∗∗^	-0.51^∗∗^	-0.46^∗∗^	0.67^∗∗^	0.83^∗∗^	0.82^∗∗^	1.00				
TB-Cr	-0.79^∗∗^	-0.27^∗∗^	0.34^∗^	-0.34^∗∗^	-0.28^∗∗^	0.64^∗∗^	0.69^∗∗^	0.89^∗∗^	0.75^∗∗^	1.00			
TC-Cr	-0.67^∗∗^	-0.180^∗^	0.19^∗^	-0.20^∗∗^	-0.15	0.43^∗∗^	0.56^∗∗^	0.59^∗∗^	0.74^∗∗^	0.75^∗∗^	1.00		
*t*_4_	-0.03	0.10	-0.14	0.13	0.13	-0.10	-0.09	-0.10	-0.14	-0.08	-0.08	1.00	
*t*_5_	0.15	0.18	-0.20^∗∗^	0.20	0.19^∗^	-0.21^∗∗^	-0.27^∗∗^	-0.23^∗∗^	-0.33^∗∗^	-0.21^∗∗^	-0.23^∗∗^	0.73^∗∗^	1.00

^∗∗^*P* < 0.05, ^∗∗^*P* < 0.01.

**Table 6:** Performance of models for detecting Qi deficiency syndrome of NSCLC based on different datasets.

Datasets	Model	AUC	Sensitivity	Specificity	F1	Precision	Accuracy
Symptom	Neural network	0.9223	0.9063	0.8286	0.8657	0.8286	0.8657
	SVM	0.9321	0.8750	0.8857	0.8750	0.8750	0.8806
	Logistic regression	0.9000	0.8125	0.8286	0.8125	0.8125	0.8209
	Random forest	0.9116	0.7813	0.8571	0.8065	0.8333	0.8209
Tongue & pulse	Neural network	0.7677	0.6316	0.6897	0.6761	0.7273	0.6567
	SVM	0.7455	0.6842	0.6552	0.7027	0.7222	0.6716
	Logistic regression	0.8022	0.6842	0.8276	0.7536	0.8387	0.7463
	Random forest	0.7314	0.5263	0.8621	0.6452	0.8333	0.6716
Symptom & tongue & pulse	Neural network	0.9401	0.9310	0.8421	0.8710	0.8182	0.8806
	SVM	0.9328	0.6552	0.9737	0.7755	0.9500	0.8358
	Logistic regression	0.9301	0.7931	0.8684	0.8070	0.8214	0.8358
	Random forest	0.9229	0.8966	0.8421	0.8525	0.8125	0.8657

## Data Availability

The datasets generated and analyzed during the current study are not publicly available due to the confidentiality of the data, which is an important component of the National Key Technology R&D Program of the 13th five-year plan (No. 2017YFC1703301) in China, but are available from the corresponding author on reasonable request.

## References

[1] Torre L. A., Siegel R. L., Ward E. M., Jemal A. (2016). Global cancer incidence and mortality rates and trends--an update. *Cancer Epidemiology, Biomarkers & Prevention: A Publication of the American Association for Cancer Research, cosponsored by the American Society of Preventive Oncology*.

[2] Torre L. A., Bray F., Siegel R. L., Ferlay J., Lortet-Tieulent J., Jemal A. (2015). Global cancer statistics, 2012. *CA: a Cancer Journal for Clinicians*.

[3] World Health Organization Cancer (2020).

[4] Liu G., Pei F., Yang F. (2017). Role of autophagy and apoptosis in non-small-cell lung cancer. *International Journal of Molecular Sciences*.

[5] Travis W. D., Brambilla E., Noguchi M. (2011). International association for the study of lung cancer/american thoracic society/european respiratory society international multidisciplinary classification of lung adenocarcinoma. *Journal of Thoracic Oncology: Official Publication of the International Association for the Study of Lung Cancer*.

[6] (2020). Chinese expert consensus on antiangiogenic drugs for advanced non-small cell lung cancer (2020 Edition). *Zhonghua zhong liu za zhi Chinese journal of oncology*.

[7] Miller K. D., Nogueira L., Mariotto A. B. (2019). Cancer treatment and survivorship statistics, 2019. *CA: a Cancer Journal for Clinicians*.

[8] Choo J. R., Soo R. A. (2020). Lorlatinib for the treatment ofALK-positive metastatic non-small cell lung cancer. *Expert Review of Anticancer Therapy*.

[9] Islam K. M., Anggondowati T., Deviany P. E. (2019). Patient preferences of chemotherapy treatment options and tolerance of chemotherapy side effects in advanced stage lung cancer. *BMC Cancer*.

[10] Chen S., Bao Y., Xu J. (2020). Efficacy and safety of TCM combined with chemotherapy for SCLC: a systematic review and meta-analysis. *Journal of Cancer Research and Clinical Oncology*.

[11] Liu R., He S. L., Zhao Y. C. (2015). Chinese herbal decoction based on syndrome differentiation as maintenance therapy in patients with extensive-stage small-cell lung cancer: an exploratory and small prospective cohort study. *Evidence-based Complementary and Alternative Medicine: Ecam*.

[12] Chen S., Flower A., Ritchie A. (2010). Oral Chinese herbal medicine (CHM) as an adjuvant treatment during chemotherapy for non-small cell lung cancer: a systematic review. *Lung Cancer (Amsterdam, Netherlands)*.

[13] Jiang M., Lu C., Zhang C. (2012). Syndrome differentiation in modern research of traditional Chinese medicine. *Journal of Ethnopharmacology*.

[14] Xia S., Zhang J., Du G. (2020). A microcosmic syndrome differentiation model for metabolic syndrome with multilabel learning. *Evidence-based Complementary and Alternative Medicine: Ecam*.

[15] Li X., Zhang Y., Cui Q., Yi X., Zhang Y. (2019). Tooth-marked tongue recognition using multiple instance learning and CNN features. *IEEE transactions on cybernetics*.

[16] Qin B., Liang L., Wu J., Quan Q., Wang Z., Li D. (2020). Automatic identification of Down syndrome using facial images with deep convolutional neural network. *Diagnostics (Basel, Switzerland)*.

[17] Pan Z., Shen Z., Zhu H. (2020). Clinical application of an automatic facial recognition system based on deep learning for diagnosis of Turner syndrome. *Endocrine*.

[18] Xia X., Chen X., Wu G. (2020). Three-dimensional facial-image analysis to predict heterogeneity of the human ageing rate and the impact of lifestyle. *Nature Metabolism*.

[19] Bing F., Shaozi L. (2018). Unsupervised clustering analysis of human-pulse signal in traditional Chinese medicine. *CAAI Transactions on Intelligent Systems*.

[20] Wang X., Zhang B., Yang Z., Wang H., Zhang D. (2013). Statistical analysis of tongue images for feature extraction and diagnostics. *IEEE Transactions on Image Processing: a Publication of the IEEE Signal Processing Society*.

[21] Kamarudin N. D., Ooi C. Y., Kawanabe T., Odaguchi H., Kobayashi F. (2017). A fast SVM-based tongue’s colour classification aided by k-means clustering identifiers and colour attributes as computer-assisted tool for tongue diagnosis. *Journal of healthcare engineering*.

[22] Zhang J., Xu J., Hu X. (2017). Diagnostic method of diabetes based on support vector machine and tongue images. *BioMed Research International*.

[23] Wood D. E. (2015). National Comprehensive Cancer Network (NCCN) clinical practice guidelines for lung cancer screening. *Thoracic Surgery Clinics*.

[24] Brambilla E., Travis W. D., Colby T. V., Corrin B., Shimosato Y. (2001). The new World Health Organization classification of lung tumours. *The European Respiratory Journal*.

[25] Micke P., Mattsson J. S., Djureinovic D. (2016). The impact of the fourth edition of the WHO classification of lung tumours on histological classification of resected pulmonary NSCCs. *Journal of Thoracic Oncology: Official Publication of the International Association for the Study of Lung Cancer*.

[26] H B (2018). Technical guidelines for clinical research of new drugs of syndrome. *Journal of Traditional Chinese Medicine*.

[27] Institute of TCM Diagnosis HUoT, Department of Internal Medicine CAoT, Department of Surgery CAoT (1997). *Clinic terminology of traditional Chinese medical diagnosis and treatment—syndromes*.

[28] Jian-feng Z., Jia-tuo X., Li-ping T. (2017). Study on the characteristics of sub-health symptoms and TCM syndrome patterns distribution in 1 754 non-disease population. *Chinese Journal of Integrative Medicine*.

[29] Schiller F., Valsecchi M., Gegenfurtner K. R. (2018). An evaluation of different measures of color saturation. *Vision Research*.

[30] Sun X., Young J., Liu J. H. (2016). Prediction of pork color attributes using computer vision system. *Meat Science*.

[31] Qi Z., Tu L. P., Chen J. B., Hu X. J., Xu J. T., Zhang Z. F. (2016). The classification of tongue colors with standardized acquisition and ICC profile correction in traditional Chinese medicine. *BioMed Research International*.

[32] Luo Z. Y., Cui J., Hu X. J. (2018). A study of machine-learning classifiers for hypertension based on radial pulse wave. *BioMed Research International*.

[33] Shi Y., Hu X., Cui J. (2021). Clinical data mining on network of symptom and index and correlation of tongue-pulse data in fatigue population. *BMC Medical Informatics and Decision Making*.

[34] Li J., Yuan P., Hu X. (2021). A tongue features fusion approach to predicting prediabetes and diabetes with machine learning. *Journal of Biomedical Informatics*.

[35] Xiang Y., Guo Z., Zhu P., Chen J., Huang Y. (2019). Traditional Chinese medicine as a cancer treatment: modern perspectives of ancient but advanced science. *Cancer Medicine*.

[36] Su X. L., Wang J. W., Che H. (2020). Clinical application and mechanism of traditional Chinese medicine in treatment of lung cancer. *Chinese Medical Journal*.

[37] Leng J., Lei L., Lei S. F., Zhu Z., Ocampo A., Gany F. (2020). Use of traditional Chinese herbal medicine concurrently with conventional cancer treatment among Chinese cancer patients. *Journal of Immigrant and Minority Health*.

[38] Zhang K., Geng W., Zhang S. (2018). Network-based logistic regression integration method for biomarker identification. *BMC Systems Biology*.

[39] Liu C., Cheng Y. (2018). An application of the support vector machine for attribute-by-attribute classification in cognitive diagnosis. *Applied Psychological Measurement*.

[40] Kong Y., Yu T. (2018). A deep neural network model using random forest to extract feature representation for gene expression data classification. *Scientific Reports*.

[41] Yu L., Chen H., Dou Q., Qin J., Heng P. A. (2017). Automated melanoma recognition in dermoscopy images via very deep residual networks. *IEEE Transactions on Medical Imaging*.

[42] Xia S., Cai J., Chen J. (2020). Factor and cluster analysis for TCM syndromes of real-world metabolic syndrome at different age stage. *Evidence-based Complementary and Alternative Medicine: Ecam*.

[43] Gu Y., Wang Y., Ji C. (2017). Syndrome differentiation of IgA nephropathy based on clinicopathological parameters: a decision tree model. *Evidence-based Complementary and Alternative Medicine: Ecam*.

[44] Kang H., Zhao Y., Li C. (2015). Integrating clinical indexes into four-diagnostic information contributes to the traditional Chinese medicine (TCM) syndrome diagnosis of chronic hepatitis B. *Scientific Reports*.

